# A Rare Case of Schistosomiasis (Bilharzia) of the Bladder in a Non-Endemic Area

**DOI:** 10.12659/PJR.901140

**Published:** 2017-07-10

**Authors:** Haresh G. Thummar, Hemen I. Vithlani, Pokhraj P. Suthar, Deepa Regina John, Nisha Thummar, Harendra Chauhan

**Affiliations:** 1 Department of Surgery, Sterling Hospital, Vadodara, Gujarat, India; 2 Department of Radiology and Imaging Sciences, Sterling Hospital, Vadodara, Gujarat, India; 3 Department of Radiology, Shri Sayajirao General (SSG) Hospital, Medical College, Vadodara, Gujarat, India

**Keywords:** Calcinosis, Endemic Diseases, Hematuria, Parasitic Diseases, Urogenital Abnormalities

## Abstract

**Background:**

Schistosomiasis or snail fever is an endemic parasitic infection caused by various trematodes of the genus *Schistosoma.* People acquire the disease through contact with water containing infected snails. It is one of the most widespread human parasitic infections in tropical and subtropical regions of the world such as Africa, South America, the Middle East, Asia and the Caribbean. In 1996, the World Health Organisation estimated that more than 200 million people living in rural areas are affected by this disease. However, the diagnosis is difficult in low prevalence areas because of a low index of suspicion.

**Case Report:**

Herein, we present a case of a 14-year old boy who had intermittent passage of blood in urine for the past 3 years. Clinical examination and initial investigations did not reveal any abnormality. Bladder schistosomiasis was suspected after contrast-enhanced computed tomography and later confirmed by cystoscopic biopsy.

**Conclusions:**

Bladder schistosomiasis is a prevalent disease in the developing countries, but in non-endemic areas diagnosis may be often missed. The diagnosis should be considered in patients presenting with sporadic episodes of haematuria who have immigrated from or travelled to areas where this disease is endemic.

## Case Report

**Figure 1:**
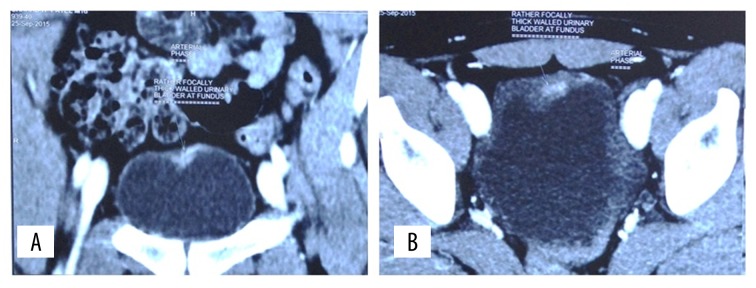
(**A**) Coronal post contrast CT image showing enhancing focal wall thickening in the fundic region of urinary bladder. (**B**) Axial post contrast CT image showing enhancing focal wall thickening in the anterior part of fundic region of urinary bladder.

**Figure 2:**
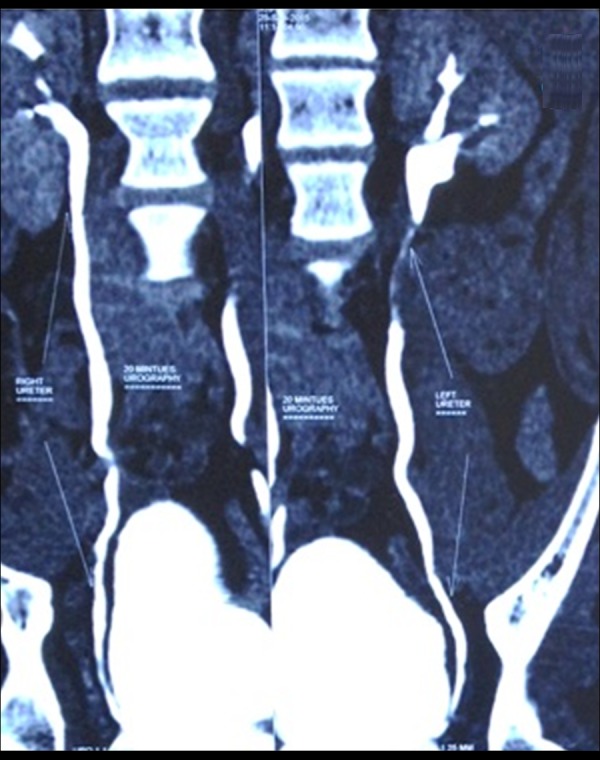
Coronal CT urography image showing filling defect in the left upper ureter; the right ureter appears unremarkable.

A 14-year-old boy presented to the outpatient department of our hospital with complaints of intermittent haematuria over the past 3 years. There was no associated pain on micturition. His birth and developmental history was unremarkable, and he was physically healthy otherwise. There was no history of trauma or rigorous exercise. No history of swimming in fresh water pool. On detailed evaluation of history, we came to know that the patient had travelled to the South African countries. Abdominal and genitourinary examination did not reveal any abnormality. His urine analysis revealed plenty of red blood cells per high power field. His coagulation profile and immunological tests gave normal results. On ultrasonography, the bladder was empty and did not reveal any significant abnormality. Both kidneys appeared normal with no signs of hydronephrosis or hydroureter. To rule out urogenital and retroperitoneal abnormalities, contrast-enhanced computerised tomography was done, which showed a focal thickening of the wall of the urinary bladder in the fundic region (Figure 1A, 1B). Coronal CT urogram revealed a filling defect in the left upper ureter (Figure 2). Urine microscopy showed eggs of Schistosoma haematobium, which is oval in shape and has a spiked end (Video 1).

**Figure 3:**
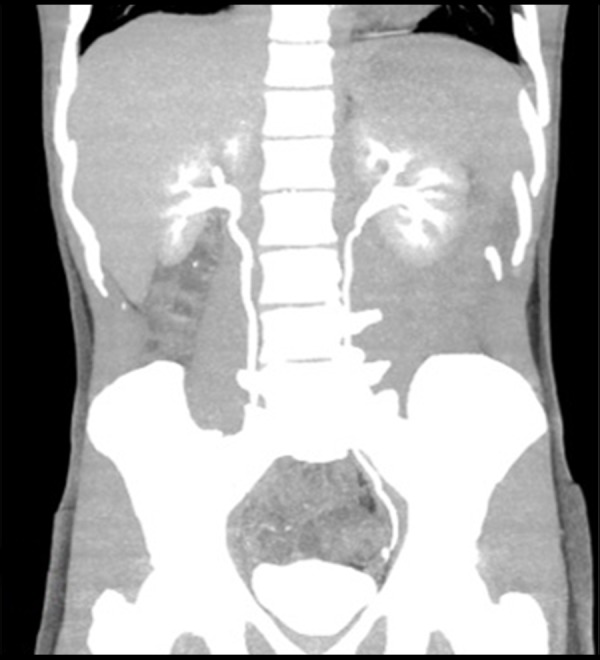
Coronal CT urography image showing no evidence of filling defect in the left upper ureter.

**Figure 4:**
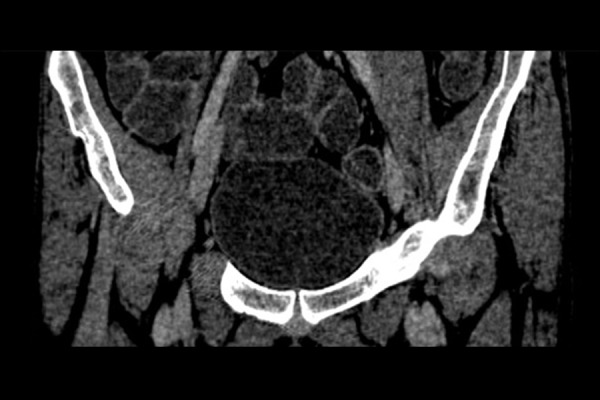
Coronal post contrast CT image showing no evidence of any enhancing focal wall thickening in the fundic region of urinary bladder.

The patient was treated with oral Praziquantel, 60 mg/kg/day in 3 divided doses, and the same dose was repeated after 4 weeks. After a full course of treatment, the patient was asymptomatic, his urine examination was normal and a CT scan did not reveal any filling defect in the left upper ureter (Figure 3). The enhancing lesion in fundus of the urinary bladder also resolved (Figure 4).

## Discussion

Schistosomiasis is a parasitic infection caused by the trematode (flat worm) *Schistosoma*. Bladder schistosomiasis is caused by the *Schistosoma haematobium* species. The larvae (cercariae), which are released from fresh water snails (intermediate host) into water, penetrate through the human skin and travel to the lungs and liver. Swimmer’s itch, characterised by a maculopapular rash, occurs 1–2 days after skin penetration, and Katayama fever with cough, malaise, weight loss and urticaria develop a few weeks later [1]. Once the larvae mature into adult worms, they travel into pelvic veins, and eggs are deposited along the bladder wall vessels and are excreted in urine [2]. The eggs incite a chronic inflammatory response with granuloma formation and also rarely cause metaplasia of the bladder epithelium resulting in squamous cell carcinoma.

Most infections occur in childhood, from the age of about 4 years and a peak age of 15–20 years [1]. Symptoms are nonspecific and include suprapubic pain, terminal haematuria and dysuria [3]. Urine cytology reveals microscopic or macroscopic haematuria and can help in differentiating glomerular and non-glomerular causes of haematuria. A definitive diagnosis is made by urine microscopy of a midday urine sample by demonstrating the eggs. Cystoscopy reveals white raised lesions in the acute phase and flattened lesions with fibrosis in the chronic stage [4]. Cystoscopic biopsy is confirmatory.

Imaging findings of bladder schistosomiasis on urography or contrast-enhanced computerised tomography include a nodular thickening of the bladder wall in the acute phase. There may be an ureteric dilatation. In the chronic phase, curvilinear calcifications, which represent multiple calcified dead eggs, are seen along the bladder wall and can be appreciated on x-rays as well as on CT. On x-rays, they are first visible at the bladder base forming a linear pattern paralleling the upper border of the pubic bone. MR urography, though more expensive, is a better imaging alternative to CT urography as it does not cause exposure to radiation, and contrast-related side effects can be avoided. However, due to a low availability of MR urography in our department, CT urography was done. An advantage of CT over MRI is that it better depicts calcified dead eggs. Differential diagnosis of bladder wall calcifications includes tuberculous cystitis, proteus infection, primary amyloidosis, neoplasm, irradiation and toxins such as cyclophosphamide. Ureteral calcifications may coalesce and extend into its entire length in a linear pattern. Later in the course of the infection, bladder wall becomes fibrotic, and the bladder contracts with a reduction in its capacity that can be appreciated on cystography. In case secondary inflammation or carcinoma develops, an enhancing mass or a focal or diffuse thickening of the bladder wall may be seen on imaging [4]. Focal disruption of linear calcifications may also favour the diagnosis of malignancy. Voiding cystourethrography is performed for detecting vesicoureteral reflux which occurs as a late complication due to ureteral fibrosis and stenosis.

Complications of schistosomiasis of the bladder include progression to calcification and fibrosis and development of squamous metaplasia and squamous cell carcinoma. Ureters may show fibrosis, calcifications and irregular dilatations due to stricture formation. Renal involvement may also be seen in late stages in the form of hydronephrosis, renal calculi and pyonephrosis. Treatment is administered by giving a broad-spectrum antihelminthic drug praziquantel that destroys the adult worms. It cures schistosomiasis in 80–90% of patients and causes a 90% reduction in egg excretion in those not cured [1]. However, the chronic fibrotic changes in the bladder wall cannot be cured. The patient should be followed up for the next 6 months for confirming eradication of eggs. Irradiated cercariae vaccine is under clinical trial [5].

## Conclusions

Symptoms of schistosomiasis can be easily missed in non-endemic areas. A thorough history and awareness of the disease can help avoid unnecessary interventional investigations and treatment delay. With appropriate clinical history, a bladder wall calcification should raise a high degree of suspicion of bladder bilharzia even with negative urinary egg test results.

### Learning points

- Schistosomiasis is associated with exposure to fresh water.
- Haematuria is the first clinical sign of genitourinary schistosomiasis which appears 10–12 weeks after infection through the skin.
- In the chronic phase, a network of dense curvilinear calcifications is noted on x-ray and computerised tomography.
- Praziquantel is the drug of choice for the treatment of schistosomiasis.

## Figures and Tables

**Video 1:**
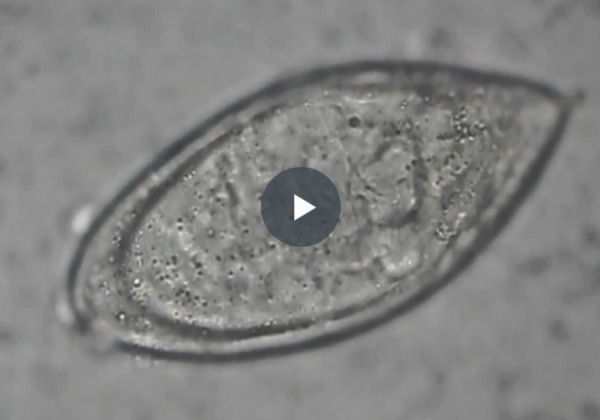
Urine microscopy shows eggs of Schistosoma haematobium, which is oval in shape and has a spiked end.
